# An Overview of the Mechanical Integrity of Dental Implants

**DOI:** 10.1155/2015/547384

**Published:** 2015-10-25

**Authors:** Keren Shemtov-Yona, Daniel Rittel

**Affiliations:** Faculty of Mechanical Engineering, Technion, Israel Institute of Technology, 32000 Haifa, Israel

## Abstract

With the growing use of dental implants, the incidence of implants' failures grows. Late treatment complications, after reaching full osseointegration and functionality, include mechanical failures, such as fracture of the implant and its components. Those complications are deemed severe in dentistry, albeit being usually considered as rare, and therefore seldom addressed in the clinical literature. The introduction of dental implants into clinical practice fostered a wealth of research on their biological aspects. By contrast, mechanical strength and reliability issues were seldom investigated in the open literature, so that most of the information to date remains essentially with the manufacturers. Over the years, implants have gone through major changes regarding the material, the design, and the surface characteristics aimed at improving osseointegration. Did those changes improve the implants' mechanical performance? This review article surveys the state-of-the-art literature about implants' mechanical reliability, identifying the known causes for fracture, while outlining the current knowledge-gaps. Recent results on various aspects of the mechanical integrity and failure of implants are presented and discussed next. The paper ends by a general discussion and suggestions for future research, outlining the importance of mechanical considerations for the improvement of their future performance.

## 1. Introduction

**Figure 1:**
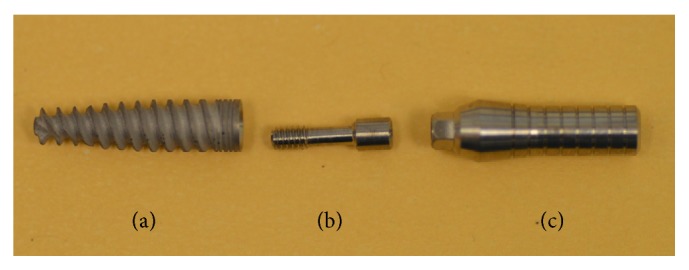
Implant components: (a) implant body, (b) abutment screw, and (c) abutment.

For the last few decades, implant dentistry has become a very popular solution to remediate the loss of teeth and durably restore both esthetics and mastication ability to (partially or fully) edentulous patients. The overall high success rate and predictability of the process, and with the relatively small percentage of complications during and after implantation, have made this procedure highly accessible to the general population. A typical dental implant, as shown in Figure 1, includes the* implant body* which is the part of the implant designed to be surgically placed into the bone. Root form implants are the common implant body form, with a screw design aimed to strongly fix the implant to the bone. The* abutment* is the part of the implant that serves to support and/or retain the* suprastructure* (i.e., fixed or removable prosthesis) [1].

Together with the high rate of success of dental implants, and with their growing use comes an increased incidence of complications, among which the mechanical ones. Like any other mechanical structure, implants are likely to fracture upon extended use. This issue is seldom mentioned or addressed in the literature, as opposed to the so-called “biological” failure to be developed in the sequel.

Therefore, this paper will survey the mechanical reliability of dental implants and address the specific issues of fracture, causes, mechanisms, and future solutions, all for a better control and performance.

## 2. About Dental Implants 

A dental implant is an alloplastic biomaterial that is surgically inserted into the jaw bone to solve functional and/or esthetic problems [1]. Most dental implants today are made from commercially pure titanium (CP-Ti grade 4) or from titanium alloy Ti-6Al-4V ELI (extra low interstitial) [1–4]. This material selection is based on the well-established properties of biocompatibility and corrosion resistance of those materials that are attributed to the native surface oxide (TiO_2_), 2–10 nm thick layer [1, 4, 5].

The success of dental implants is largely attributed to what is known as “osseointegration,” a term originally defined by Branemark in 1952. Osseointegration implies an anchorage mechanism, whereby artificial components can be reliably and predictably incorporated into living bone, and that this anchorage can persist under all normal loading conditions [6].

Two main parameters contribute to a successful process of osseointegration. The first is the implant's* surface characteristics*, and the second, of a more macroscopic nature, is the* implant design* which enables primary stability needed for the biological process of osseointegration to occur [7]. Implant movements, without primary stability, even at the micrometer range, may negatively influence osseointegration and bone remodeling by forming fibrous tissues, thereby causing bone resorption at the bone-to-implant interface [8].

### 2.1. Implant Surface Characteristics

The effect of surface characteristics on the biological reaction and on bone to implant contact has been studied extensively in the dental implant community. Surface treatments increase the active surface area and allow a firmer mechanical bond to the surrounding tissues [8]. In addition, surface topography leads to faster and stronger bone anchorage and may confer improved stability during the healing process, thus allowing earlier loading (dental terminology for usage) of the implant [9, 10]. Average height deviation parameters (*R*_*a*_ and *S*_*a*_) between 1 and 2 *μ*m, which define a “moderately rough surface,” were found to be optimal for a successful osseointegration process [9–12].

A great variety of surface treatments exist today, in order to achieve a desired degree of surface roughness. The different surface modifications can be divided into six types: as-machined, plasma spray and laser peening (laser surface treatment, LST), acid etching, grit blasting followed by acid etching, anodizing, and biomimetic coating. Among those,* grit blasting* is one of the most common dental implant surface treatments [10, 12]. Blasted surface roughness with *S*_*a*_ values ranging from 0.6 to 2.1 *μ*m is deemed ideal for the implant's osseointegration [9]. During this process, implants are blasted with air-propelled hard ceramic particles (Al_2_O_3_, TiO_2_, and Ca_2_P_2_O_7_) [11]. Depending on the size of the ceramic particles and their velocity, different surface roughness levels can be produced on the implant's surface.

### 2.2. Implant Design

The goal of a successful implant design is to best anchor the implant into the bony ridge. Most implants today are “root form” implants with a screw design aimed to strongly fix the implant to the bone [1, 3] (Figure 1). Additional design features, such as thread depth and width, thread pitch, thread geometry, and helix angle, can all be optimized (at least in principle) by implant manufacturers to initially support the implant and to hold it in place. At this stage, one should keep in mind that dental implant design is dictated by the same considerations, which are characteristic of any other structural design project.

## 3. Complications in Implant Dentistry 

### 3.1. Biological

Complications associated with dental implants can lead to implant failure and to its loss. Implant failure can be divided into two categories.

The first, early failures, occurs* no later than 6 months* after implantation or before the implants are loaded. The second, late failures, occurs beyond the initial 6-month period after implantation [13–16].

Early failures are mainly of a* biological* nature. In that case, the process of osseointegration did not succeed due to various reasons, such as surgical trauma, infection, and implant micromovements as a result of premature loading. Over 50% of implant losses are viewed as early losses [14, 15, 17].

Late failures are categorized with respect to their cause. First are the biological causes, such as progressive loss of bone support as a result of infection or inflammation known as peri-implantitis [13–16]. About 50% of implant losses occur at late stages, for the most during the first year after loading, due to loss of bone support [14, 15, 17]. Snauwaert et al. [18] report that most of the late biological failures occurred only 1 year after loading (60%), and the rest from the second year on.

### 3.2. Mechanical

**Figure 2:**
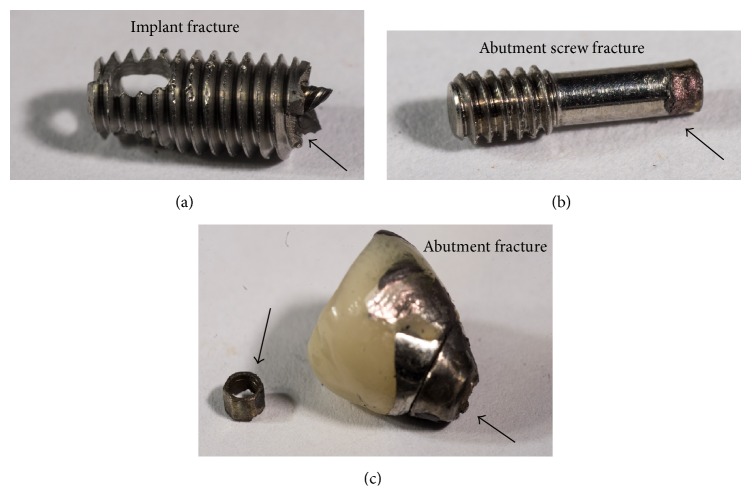
Macroscale photographs of retrieved fractured implants and implant's components: (a) fractured implant, (b) fractured abutment screw, and (c) fractured abutment.

The second cause for implant loss is related to* mechanical complications*. This term covers mechanical damage in general, whether to the implant, its components, or the suprastructure. Implant loss, which relates to mechanical complications, includes implant fracture, abutment screw fracture, and abutment fracture (Figure 2).

Implant fracture is considered a severe complication requiring extraction of the implant and its supporting bone [18–21]. Since this review article is concerned with mechanical reliability of dental implants, the issue of mechanical failure will be developed next in detail.

A series of recent systematic reviews, based on clinical studies with 5- and 10-year follow-up periods, reported a high incidence of such mechanical complications [16, 21, 22], with a 5-year complication rate for a total number of mechanical complications ranging from 16.3% to 53.4% [22].* Screw fracture* is most commonly encountered with a 5- and 10-year rate of 9.3% and 18.5%, respectively. Implant fracture is considered as a severe but rare complication, with a 5-year complication rate of up to 4% [22]. A long-term retrospective study on the status of 1325 implant, after a follow-up time of 29 years was carried out by Dhima et al. [23]. Those authors reported that they observed more mechanical complications than biological ones. According to this study, more than half (58%) of the implants experienced* at least* one mechanical complication. This study also showed that mechanical complications occur well after the biological ones. While a mean time of 5 years is reported for biological complications, it becomes 7.6 years for mechanical complications. Here too, the most common mechanical complications consist of screw (8.5%) and abutment fracture (5.5%).

Considering specifically implant fracture now, Manor et al. [17], who characterized a large number of surgically removed implants, reported fracture for 6% of them. Pommer et al. [24] recently published a meta-analysis on the incidence of implants' fracture, reviewing clinical studies that reported such fractures. These authors concluded that the incidence of implant fracture jumps to 2.8% after a follow-up time of 8.3 years. Yet, most of implant fracture cases reported in this study occurred just after 4.1 ± 3.5 years. Those results emphasize the importance of the follow-up time on the occurrence of implant fracture.

To summarize, all the studies, dealing with implant loss and implants complication rates, have clearly pointed out that mechanical complications (especially* implant fracture*) do actually occur frequently after relatively long follow-up time periods. Mechanical complications occur significantly later and more frequently than biological complications, and yet, their severity is much more pronounced because of the complexity of treatment that ensues.

Here, one should note the distinct character of the mechanical failure which is of a* time-dependent nature*, as opposed to monotonic failure, the latter meaning immediate overload failure upon service. Monotonic failure means one of the two (or both) of the following: poor design and/or abnormally high loads. Since such failures are not reported in the literature, the time-dependent nature of implants' mechanical failure reminds immediately two classical time-dependent failure mechanisms, fatigue and/or (stress) corrosion [25, 26]. This subject will be elaborated upon in the sequel.

Mechanical complications can be related to the nature and amplitude of the mastication loads and hence implant stresses. The type of restoration supported by the implants (removable or fixed prosthesis) may influence both the amplitude and nature of the loads transmitted to the implant, as can be understood from Berglundh et al. [14]. Occlusal loads magnitude is in itself a key factor for implants loading. Among those loads, parafunctional habits such as bruxism and clenching may increase the implant/prosthesis stress, leading to early occurrence of mechanical complications, according to De Boever et al. [27].

## 4. Failure Analysis in Implant Dentistry 

Failure analysis is the process by which (in the mechanical context) a broken component is examined using various techniques to determine the causes and fracture mechanisms on the one hand but also provide guidelines and recommendations to avoid future failures on the other hand.

Examining the fracture surface of (retrieved) fractured dental implants and implant components is the optimal procedure to assess the cause(s) and mechanism(s) of fracture [28]. Detailed failure analyses of retrieved fractured dental implants are quite rare in the dental and in the biomechanical literature alike. It should be noted that the extraction of fractured implants is a complex surgical procedure, so that most of them are left in the alveolar bone. One should also note that the fracture surface of the implants, which is essential for fracture analysis, is often damaged heavily or even destroyed as a result of the surgical procedure. Such destruction complicates significantly the identification process of the fracture cause(s) and mechanism(s), which is based on scanning electron fractographic analysis. With that, a few published articles [29–33] that examined the fracture surface of retrieved fractured dental implants identified the probable mechanisms responsible for mechanical failure. Most of those studies [29–31] identified* metal fatigue* [25] as the main failure mechanism on implants made of CP-Ti only. Yet, the cause(s) for fatigue crack initiation and the crack nucleation site(s) could not be clearly identified, leaving this issue unsolved.

**Figure 3:**
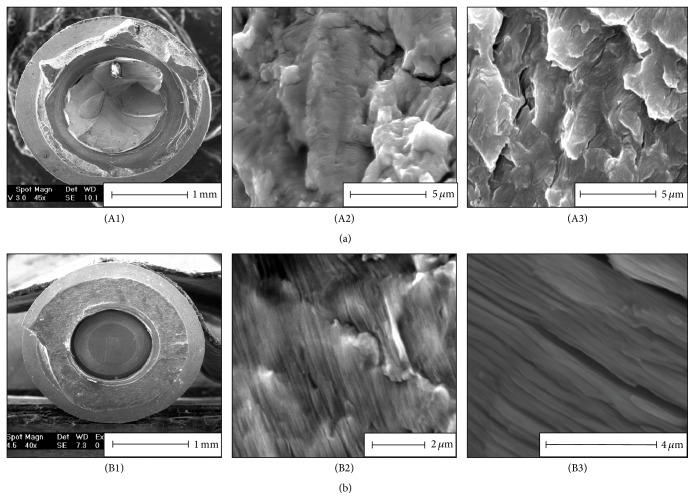
Failure analysis of retrieved fractured Ti-6Al-4V implant (a) and CP-Ti implant (b). (A1) and (B1) are macroscopic views of the fracture surface of the implant. (A2) and (B2) show fatigue striations on retrieved fractured implants. (A3) and (B3) show fatigue striations on dental implants fractured in laboratory conditions in room air. Note the high resemblance of the in vivo and in vitro fracture surface topographies.

Recently, Shemtov-Yona and Rittel [33] performed a detailed systematic failure analysis on 10 retrieved CP-Ti and 8 Ti-6Al-4V fractured dental implants and implant parts. The failure analysis confirmed, through a comparison between fracture surfaces of the retrieved fractured implants (in vivo) and fracture surfaces of implants fractured in lab conditions (in vitro, exemplar testing), that the operating fracture mechanism was indeed metal fatigue, which obviously relates to the repetitive character of mastication loads (Figure 3).

In addition, the analysis showed (based on fracture-mechanics considerations) that implants and their parts might be broken at relatively low cyclic load levels, of the kind that matches the relatively low load levels generated during mastication [34].

Additional work by Shemtov-Yona et al. [35], consisting of surface examination by scanning electron microscopy of fractured implants, revealed numerous secondary cracks in the vicinity of the main crack (fracture plane). These secondary cracks did not lead to the final fracture, but they did reveal the relationship of the secondary cracks to the surface topography (roughness).

The added value of those works lies in the systematic identification and characterization of the* fatigue crack growth mechanism* [33] that was suggested in earlier works but was not entirely supported. Likewise, in the second work [35], the probable origin of the fatigue cracks was identified, whereas the literature so far had not addressed this issue.

**Figure 4:**
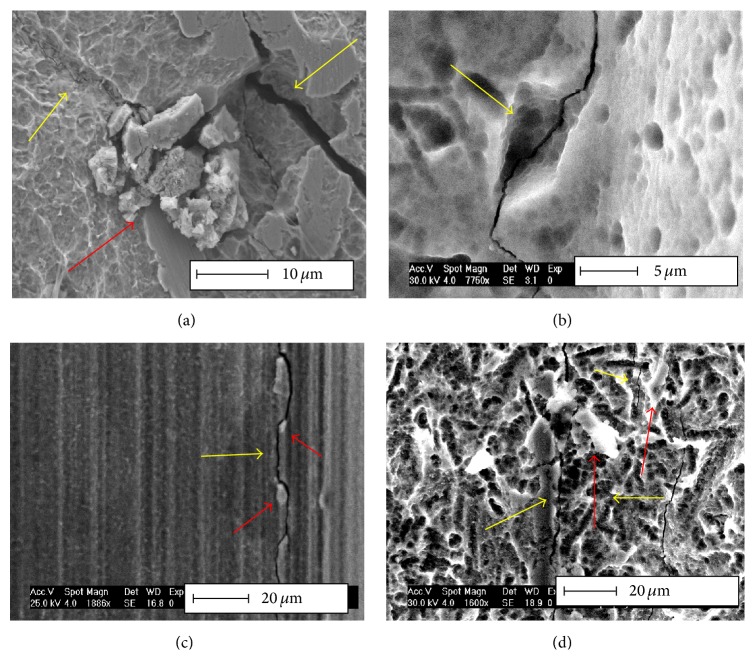
Typical surface topography of dental implants that were fractured and retrieved from the oral cavity. Note the numerous secondary cracks, arrowed in yellow. Embedded foreign particles (arrowed in red) are associated with the crack path; (a), (b), and (d) originate from grit-blasted and etched implants, while (c) originates from an as-machined surface. Reprinted with permission from [35].

More specifically, the systematic examination of the implants' surface revealed a definite connection between embedded ceramic particles, which resulted from the various surface treatments, and the generation of cracks, as shown in Figure 4. The relationship between the initial surface condition and implant late fracture is presented next.

## 5. Surface Treatments and Their Influence on the Mechanical Durability of Dental Implants 

The effect of grit blasting treatments, one of the most common surface treatments in biomedical application, on fatigue performance of CP-Ti and titanium alloys has been studied in several instances. Baleani et al. [36] studied the effect of grit blasting treatments with alumina particles on the fatigue endurance strength of Ti-6Al-4V. This study showed that grit blasting of Ti-6Al-4V reduces its endurance limit by 35–40%. This observation was linked to stress concentrations and surface roughness issues.

On the other hand, a study by Pazos et al. [37] on CP-Ti showed that the fatigue behavior of a blasted surface and of an as-machined surface is similar even though fatigue crack nucleation, on grit blasted specimens, resulted from stress raisers notches which were created by the alumina particles. To explain this result, the authors invoked the introduction of beneficial compressive residual stresses that balanced to some extent the adverse effect of the stress raisers.

Leinenbach and Eifler [38] compared the fatigue performance of grit blasted and polished Ti-6Al-4V cylindrical specimens. In this work, the endurance limit of the grit blasted material was diminished by 25%. The grit blasted specimen, after fatigue testing, was investigated using a scanning electron microscope (SEM). The investigation revealed the formation of microcracks in the vicinity of the embedded alumina particles. The study concluded that the fatigue cracks initiated at remaining ceramic particles or in sharp grooves where large stress concentrations exist.

The effect of grit blasting treatment, with alumina particles, on the mechanical properties of Ti-6Al-4V subsurface area was also examined by Multigner et al. [39]. The study showed that the treatment severely deformed the material surface, thus creating sharp ridges and crack-like defects. The authors concluded that the decrease in fatigue strength is the result of surface defects which were created during the grit blasting of the Ti-6AL-4V and acted as severe notches. It is important to note that none of the above-mentioned studies did consider the parameters of the treatment, such as particle size, material, and impact velocity. Therefore, the reported contradictory observations might simply result from the fact that the initial surface condition was different in each research.

Gil et al. [40] evaluated the effect of surface treatments on the fatigue behavior of commercial dental implants. The study compared the fatigue behavior of grit-blasted implants to that of machined implants made of CP-Ti, through the construction of S-N curves. The study showed that grit blasting of CP-Ti dental implants improves their fatigue life due to the layer of compressive residual stresses that is formed.

Ayllón et al. [41] compared the fatigue behavior of CP-Ti implants with and without grit blasting surface treatment. The results of the study showed, on the contrary, that the surface treatment actually reduced the implant's fatigue limit by approximately 12%.

From the above literature survey, it appears that contradictory conclusions are drawn regarding the beneficial or detrimental effect of the grit blasting surface treatment on the fatigue performance of implants. However, as stated earlier, while those works concentrated on the fatigue performance from a total-life point of view, the issue of the factors responsible for the nucleation of microcracks was not really looked at in detail.

**Figure 5:**
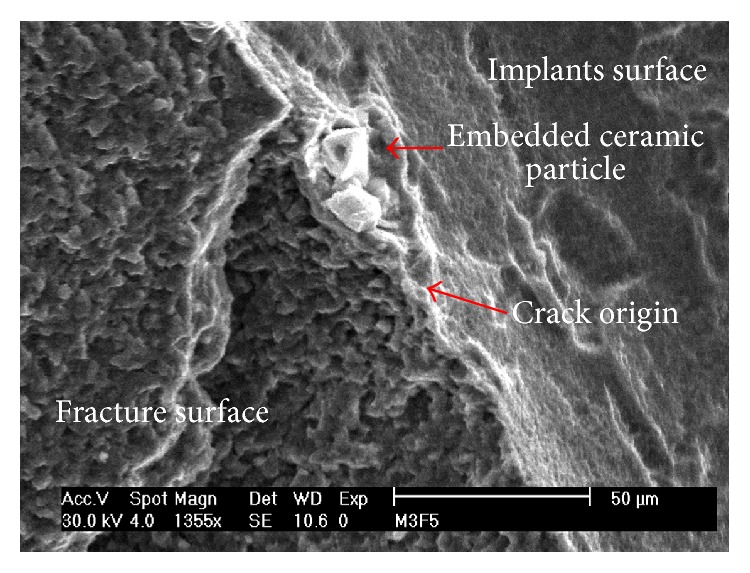
The involvement of embedded ceramic particle in fatigue crack initiation in dental implants. The origin of the fatigue crack was traced to the implant surface. The connection to the embedded particle left behind during the surface treatment can be easily observed.

Shemtov-Yona et al. [35] studied the influence of surface preparation treatments of dental implants on their potential (mechanical) fatigue failure, with emphasis on grit blasting. The study showed that embedded alumina particles, which are related to an uncontrolled grit blasting process, can induce significant surface damage which accelerates the fatigue failure mechanism and leads to rapid fracture (Figure 5). Uncontrolled grit blasting is to be understood in this context as a treatment whose outcome is the strong embedding of ceramic particles into the implant's surface, which often comes along with the generation of microcracks in the vicinity of the impact. This means that, while the goal of the surface treatment is both to roughen and harden the implants surface to provide improved osseointegration and fatigue properties, respectively, it appears that excessively high impact velocities are likely to create surface microcracks which are the nuclei of future fatigue cracks.

In other words, a process that was initially developed as a* beneficial* process essentially for osseointegration may turn to be* detrimental* if carried out in an uncontrolled fashion, as it may introduce surface defects. This is all the more so severe when titanium and its alloys are notorious for being highly resistant to the development of fatigue cracks [42], and in this case, the seeds for those cracks are readily supplied as a result of the ceramic particles impact.

## 6. Implant Design Considerations: Sizing

Stress concentrations might be generated along the implant geometry due to faulty design parameters. These stress concentrations can also accelerate nucleation of fatigue cracks. Khraisat et al. [43] assessed the effect of joint design on fatigue strength and failure mode of 2 implant systems. The first implants are made of CP-Ti grade 1, 4 mm wide, with external joint configuration. The second implants are made of CP-Ti grade 4, 4.1 mm wide, with internal joint configuration. The study showed superior fatigue performance to the implants with internal joint configuration and identified the crucial point to failure on the failed external joint configuration group, at the junction between the unthreaded and threaded parts of the abutment screw. This study emphasizes the importance of different implant design on different fracture locations, in relation to stress concentrations created along the implants.

Examining the implant diameter as a design parameter important for the implants fatigue life was evaluated by Quek et al. [44]. The study investigated the fatigue performance of 3 different widths of single-tooth implants and abutments. The test results showed that the implant body is a possible fatigue failure location in narrow implants (3.3 mm) during cyclic loading, compared to a superior fatigue performance of the 5 mm wide implants. However, this study was based on only 5 implants, with only one level of applied load, which is obviously of a limited nature.

**Figure 6:**
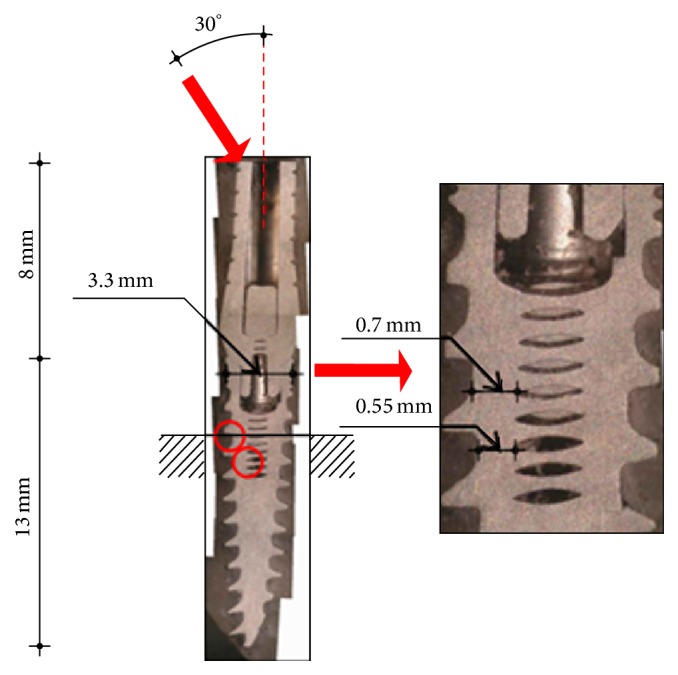
Metallographic section from in vitro fatigue testing of 3.3 mm implant diameter. The length and width of the implant and abutment are indicated. The upper red arrow indicates testing force applied to the implant abutment and the force direction at an angle of 30° off-axis. The red circles indicate the different fracture location found for this implant diameter. The magnified picture shows the fracture location and the corresponding metal width at the fracture location. Reprinted with permission from [46].

Shemtov-Yona et al. [45, 46] recently evaluated the total fatigue life of 3 different implant diameters. In the failure analysis that was presented, it was shown that the various failure loci observed, which probably caused fatigue crack initiation, were all connected to some sort of* stress concentration*. The latter is caused by geometrical (design) effects. The research demonstrated that narrow diameter implants had a profound effect on implants fatigue performance. Proper implants' design is crucial to ensure long-term fatigue performance for dental implants. The combination of sharp notches (thread) and narrow metal cross-section derived from the implant's diameter affect negatively the implants fatigue performance (Figure 6).

All those results suggest that implant design and optimization are highly desirable and may delay mechanical failure, if carried out properly by reducing stress concentrations and increasing wall thickness wherever necessary.

Among the most suitable design tools, the finite element method (FEM) [47, 48] offers the highest flexibility in terms of both geometrical and material parameters variations and their influence on the implant stresses. While numerous finite element studies of dental implants have been carried out to date, they will not be included in the present review, which deals essentially with fracture and not stress analysis of dental implants.

## 7. The Likelihood of Dental Implant Fracture

As mentioned in the Introduction, the accepted paradigm is that implant fracture is an unusual event and therefore seldom reported or discussed in the literature, while the actual picture is with the manufacturers and practitioners alike. In fact, many implants are extracted from the jawbone for biological complications that were discussed previously, and none of those is actually broken. On the other hand, structural health monitoring of dental implants, a process that is increasingly developed for, for example, aeronautical structures, is virtually unknown in the dental community. One may argue that the early detection and monitoring of very small cracks in in vivo implants are extremely difficult to perform, if not impossible, due to inherent limitations of the current nondestructive methods.

**Figure 7:**
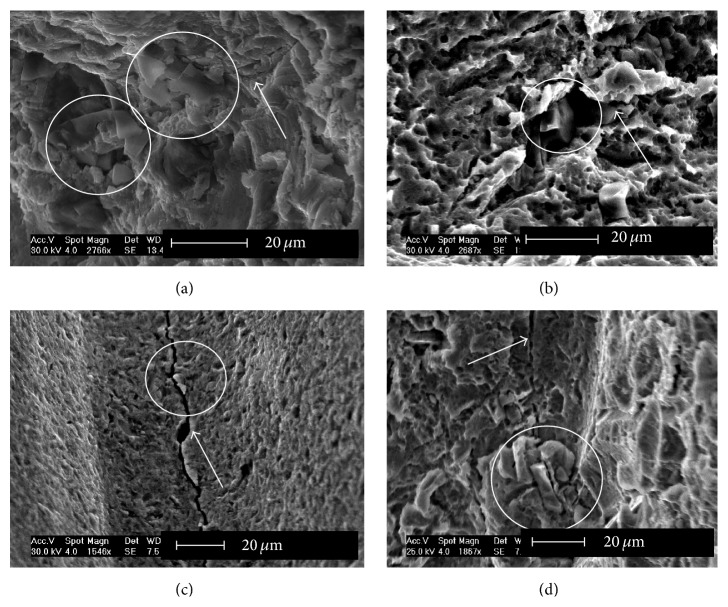
Embedded foreign particles on full cracks and crack-like defects, as identified on grit-blasted (with or without etching) implants. The white arrows mark the defects (full cracks or crack-like defects) and the white circles mark embedded foreign particles. Reprinted with permission from [49].

As a result, a standing issue is whether implanted structures are deteriorating without the knowledge of the practitioner until they break in a catastrophic manner. Shemtov-Yona and Rittel [49] addressed this issue for the first time and brought a preliminary answer to this question. Those authors interrogated systematically a cohort of 100 dental implants that were all extracted from the jawbone for biological reasons, while none of them was actually broken or even visibly damaged to the naked eye. Those implants were collected at random from 4 clinics, without any clinical or otherwise information on the patients, the implants, and their duration of service until extraction. Yet, by carefully scanning the overall surface of each and every implant, the following facts came to light. Over 60% of the scanned implants contained both defects that are analogous to microcracks and fully developed cracks. In addition, a high incidence of embedded particles was observed in close relation with the defects (Figure 7). This further strengthens the role played by the particles upon defects generation, some of which can later evolve into full cracks. One important outcome of that study was that, in those implants, the vast majority of cracks was associated with ceramic particles, indicating that no new cracks formed during service, so that future fracture, had it been allowed to develop, would have occurred from the preexisting cracks.

In the retrieved sample, they observed that the CP-Ti implants contained more defects and cracks than the Ti-6Al-4V ones. This emphasizes the importance of material selection considerations.

But all in all, this study revealed an intriguing fact: since all the flawed implants were extracted at an early stage for biological reasons only, the preexisting microcracks could not mature into macroscopic cracks causing final fracture. This observation can suggest that once the issue of biological failure will be much better or fully controlled, the issue of mechanical failure will become the prime source of complications for dental implants, which at that stage does not come often into play due to early implant extraction.

## 8. Discussion and Directions for Future Research

As shown in this review article, the issue of mechanical reliability in implant dentistry is a significant one. All published failure analysis studies on fractured implants have shed light on the causes for mechanical complications and identified the main failure mechanisms that govern the process.

Studies dealing with mechanical reliability of dental implant have succeeded in bringing to awareness the fact that implants and their components' fractures are possible and realistic complications that are bound to occur with the growing and prolonged use of implants. That is why emphasis should be put on striving not only to improve the osseointegration process, but also to preserve and maintain the implant's integrity from a mechanical point of view as well.

In order to consolidate the preventive measures for dentists who are placing and using implants to rehabilitate missing teeth, additional clinical information is needed that will link the biological/clinical data and the possible mechanical fracture that probably will be generated. In fact, as of today, this potential biological-mechanical connection has not really been explored by the dental community.

Therefore, the main points that arise from this review can now be summarized as follows, with some perspective on issues that need new or additional research effort.

### 8.1. Fatigue and Surface Condition

First, it should be noted that failure analysis studies have clearly shown that fatigue is the main failure mechanism, which leads to implants final fracture. Fatigue can be considered a multiphase failure process in which cracks are being generated (crack initiation) and then grow steadily to the end catastrophic result [25, 26].

The involvement of surface roughness and embedded foreign particle on fatigue crack initiation has been clearly identified. As sand blasting and etching are the most common implant treatment today, its clear advantage on the process of osseointegration is well documented, but there is no systematic work on the blasting treatment parameters (blasting pressure, particle size, and velocity) that will prevent the deleterious generation of fatigue cracks due to various stress concentrations on the implants surface. In parallel, one should make sure that the selected set of parameter will not hamper the short term biological success of the implants. Recommendations should be issued as to the optimal grit blasting parameters that will yield the designed surface parameters without affecting its integrity.

Yet, there is an obvious need for alternative surface roughening treatments, ideally without the use of aggressive and abrasive methods that may damage the surface and contaminate it with foreign particles, whose biological effect is not fully assessed yet. All that, of course, without lowering the probability for a successful osseointegration.

### 8.2. Fatigue and Intraoral Environment

Next, one should keep in mind that among the factors that affect fatigue crack initiation and propagation, one should consider the (intraoral) environment. This environment is constantly changing, both in chemical composition and load frequency. Implant dentistry needs to identify the various contents and elements that might jeopardize implants reliability and affect their endurance to fatigue crack propagation. Along with that, methods must be found that can simulate the oral environment along with its complex parameters. It is meant here that an “artificial oral cavity” should be developed to perform reliable tests that are as close as possible to the in vivo reality.

### 8.3. Biomaterials Selection and Testing

The field of biomaterials and material selection is a pivotal one, not only to the biological reaction of materials to the body but also to the mechanical integrity. There must be constant search for new biocompatible materials that will feature an excellent fatigue performance with both late crack initiation and slow crack propagation.

Laboratory or in vitro testing is usually applied to study the mechanical behavior and properties of dental implants. However, in vitro testing cannot be reliably extrapolated to the in vivo behavior of biomaterials, because the conditions of the oral environment cannot be simulated. The current standard recommendations [50] request testing of a minimal sample size in room air. It appears that those recommendations suffer from severe limitations. Firstly, the recommended sample size is way too small to draw any meaningful statistical conclusion as to the implant's cyclic performance, noting that statistical spread of the measured fatigue life is inherent to the field of fatigue. Moreover, testing is also practiced under harmonic loading conditions, according to the spirit of the traditional S/N curve. It would be much more realistic to test dental implants under* spectrum loading*, as routinely done in structural engineering applications, and develop accordingly a recommended testing protocol.

### 8.4. Structural Health Monitoring of Dental Implants

As of today, there is absolutely no way to detect small (fatigue) cracks in vivo, on dental implants. As a result, one cannot assess the state of damage of an implant once it is in place until significant complications occur. The current technique used in dental imaging, and not for this purpose, is essentially based on X-ray radiography. This technique has inherent limitations on the size of the flaws it can detect, so that existing cracks will go virtually undetected by the clinician. Yet, with the current developments in the field, including the high-resolution levels that are achievable with microcomputerized tomography (CT), it seems that the sought after detection is perhaps around the corner. One should therefore strive to develop and implement a technique that allows monitoring the development and evolution of small cracks in implants, such as to control the fracture process and avoid that it reaches its catastrophic end, and in addition will diagnose if there is an effect on the living tissue, as mentioned earlier. This field of engineering, called “structural health monitoring,” is constantly developing and should also be considered seriously in implant dentistry as one more advanced diagnosis tool.

Until such technological progress is achieved, it would be interesting to repeat the work of Shemtov-Yona and Rittel [49], who characterized the state of damage in retrieved dental implants, but this time with full clinical history and detailed documentation on the retrieved implants. Such a study would definitely help to better determine the evolution of mechanical damage over the years, which could serve to establish guidelines for dentists placing implants. It would also help in setting time limits for the onset of development of damage in its various forms, as well as its statistical average evolution over time. Such information would be quite useful to provide a realistic estimate of the average life expectancy of a dental implant, together with the scheduling of preventive dental examinations.

This remark also relates to our previous remark on the possible mechanical-biological coupling. Such a study technique could be implemented to study the connection and relation between the biological failure and the mechanical one and elucidate standing issues related to this possible dependence.

### 8.5. Advanced Modeling of Dental Implants

As of today, there are very powerful tools that are routinely used to perform mechanical analyses of structures. The most popular is of course the finite element method. This vast subject has not been developed in the review but will be briefly discussed here in a specific context. While the basic mechanical properties of the metals used for implants are quite well known, less is known, as mentioned, about their behavior in the oral environment since the latter is so diversified. In parallel, simple constitutive models that relate the jawbone evolution and reaction to mechanical stresses are not readily available. Yet, if a more representative model is sought, one should consider the triangle formed by the mechanical properties of the metal, the physicochemical interaction with the environment, and finally the mechanobiological response of the bone, keeping in mind they all interact so that the problem at hand is highly coupled. It is believed that with the advent of such advanced models, a wealth of numerical experiments will become possible that will save considerable amounts of money and time to the research and manufacturing communities alike.

Finally, from the present survey and the outline of several pending issues, it appears that the mechanical reliability of dental implants should be considered a truly interdisciplinary field, involving both engineering and dentistry. Since the pending issues are well defined, and many of the required techniques and know-how are readily available in each community, one can expect that future joint research in the field will contribute significantly to improve the mechanical reliability of dental implants.
